# Variations in enamel damage after debonding of two different bracket base designs: An in vitro study

**DOI:** 10.15171/joddd.2018.009

**Published:** 2018-03-14

**Authors:** Mohammad Hossein Ahangar Atashi, Amir Hooman Sadr Haghighi, Parastou Nastarin, Sina Ahangar Atashi

**Affiliations:** ^1^Department of Orthodontics, Faculty of dentistry, Tabriz University of Medical Sciences, Tabriz, Iran

**Keywords:** Damage, debonding, enamel, orthodontic bracket

## Abstract

***Background.*** Bracket base design is a factor influencing shear bond strength. High shear bond strength leads to enamel crack formation during debonding. The aim of this study was to compare enamel damage variations, including the number and length of enamel cracks after debonding of two different base designs.

***Methods.*** Eighty-eight extracted human premolars were randomly divided into2 groups (n=44). The teeth in each group were bonded by two types of brackets with different base designs: 80-gauge mesh design versus anchor pylon design with pylons for adhesive retention. The number and length of enamel cracks before bonding and after debonding were evaluated under an optical stereomicroscope ×40 in both groups. Mann-Whitney U test was used to compare the number of cracks between the two groups. ANCOVA was used for comparison of crack lengths after and before debonding in each group and between the two groups.

***Results.*** There was a significant increase in enamel crack length and numbers in each group after debonding. There was no significant difference in enamel crack numbers after debonding between the two groups, whereas the length of enamel cracks was significantly greater in anchor pylon base design after debonding.

***Conclusion.*** Bracket bases with pylon design for adhesive retention caused more iatrogenic debonding damage to enamel surface.

## Introduction

Brackets are manufactured in various designs.Variability in design includes bracket material, prescription, base size, ligation type and retentive base design.^1,2^ There are recommendations in the literature for enhancement of bracket mechanical retention on tooth surface by altering the retentive base design in order to prevent bond failure during orthodontic treatment.^3^ However, there is no evidence exploring probable undesirable side effects of increased mechanical retention between the bracket and adhesive.

Bracket base design might affect bracket‒adhesive retention, which affects the type of bond breakdown.^4^ During debonding procedure, bond failure may occur at: 1) between the bracket and the adhesive interface; 2) between the adhesive and enamel interface; or 3) within the adhesive.^4^ In cases of bond failure at adhesive‒enamel interface, there is a higher risk of enamel damage due to the existence of a micromechanical bond between the adhesive and enamel,^5,6^ which might result in enamel cracks.^7^ Enamel cracks in the longterm might lead to tooth fracture, demineralization and caries development or esthetic problems.^8^ Thus if the bracket base design affects the type of bond failure, selection of brackets with various base designs might have an important role in the development of iatrogenic damages, including enamel cracks after debonding. Several authors evaluated the influence of bracket base design variations on debonding characteristics.^3,9,10^

Wang et al^3^ concluded that circular concave base design produced higher bond strength compared to mesh-base designs, withlarger mesh spacing, resulting in higher bond strength. Gibas et al^11^ found higher shear bond strength in brackets with pylon base design compared with mesh-design brackets. Sharma-Sayal et al^12^ demonstrated that base design affects bond strength and SPEED active self-ligating brackets (Strite Industries, Cambridge, Ontario, Canada) with 60-gauge micro-etched foil mesh base and an integral undercut machined base provided higher bond strength values.

Considerable research hasindicated that base design can influence bond strength; therefore, base design might have a role in enamel stress absorption and producing iatrogenic debonding side effects on enamel, including enamel cracks which are the clinical sign of debonding damage on tooth surface. No study has been undertaken to evaluate variations in crack development due to base design.^3,10,11^

Of all thedifferent base designs available on the market, mesh design is the most common and popular;in addition, another advanced base design withanchor pylons instead of mesh, for adhesive mechanical retention, is available and employed by orthodontists.

The aim of the current study was to compare the length and number of enamel cracks after debonding with the use of two different base designs, including retentive pylons and mesh design, and also to determine the amount of adhesive remaining on tooth surface after debonding, which indicates the amount of unfavorable bond failurebetween the adhesive and enamel surface. Therefore this study aimed to reveal the possible importance of bracket base design in the development of enamel damage in order to provide guidelines on selection of a base design with less damageon enamel surface during debonding.

## Methods

### Samples

In this in vitro study, 88 extracted human premolars werecollected from patients undergoing orthodontic treatment after informed consent. Sample size determination was performed using G. power 3.1 software, considering crack number significant difference=1-1.5, mean difference=1.2 units, α=0.05 and power=80%. The teeth were stored in 0.1% thymol solution for 7 days at room temperature to prevent bacterial growth and dehydration. The samples were subsequently immersed in 4°C distilled water until bonding of brackets, which was replaced weekly for less than 3 months.^5,8,13^

The inclusion criteria consisted of intact buccal surface, no enamel lesions or caries and no history of chemical agent application or fluorosis. The teeth were examined by transillumination for inclusion.^5,13-15^

The samples were randomly divided into two groups (N=44). Each tooth had a numerical codeandwasmounted in self-cured acrylic resin at CEJ.

**Figure 1:**
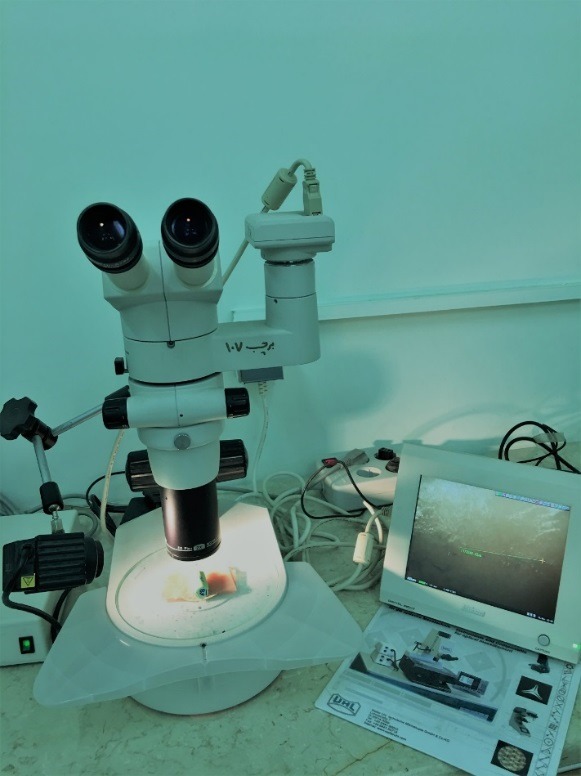
Stereomicroscope connected to digital camera.

All 88 sampleswere examined under an optical stereomicroscope (Nikon, Japan) at ×40,connected to a digital camera (Nikon, Japan) (Figure 1) with the ability of linear measurements for evaluation of the number and length of primary enamel cracks. All the teeth were evaluated atthe same distance from the buccal surface to the lens.^16^

### Brackets

**Figure 2:**
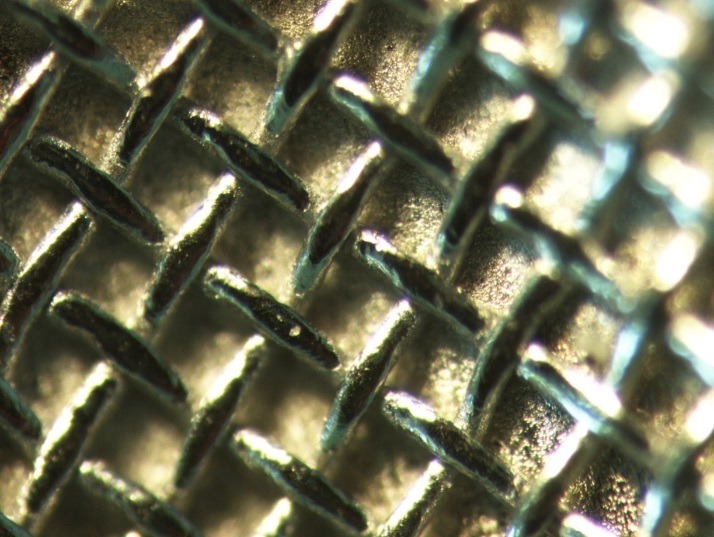
Mesh base design at ×40 magnification.

**Figure 3:**
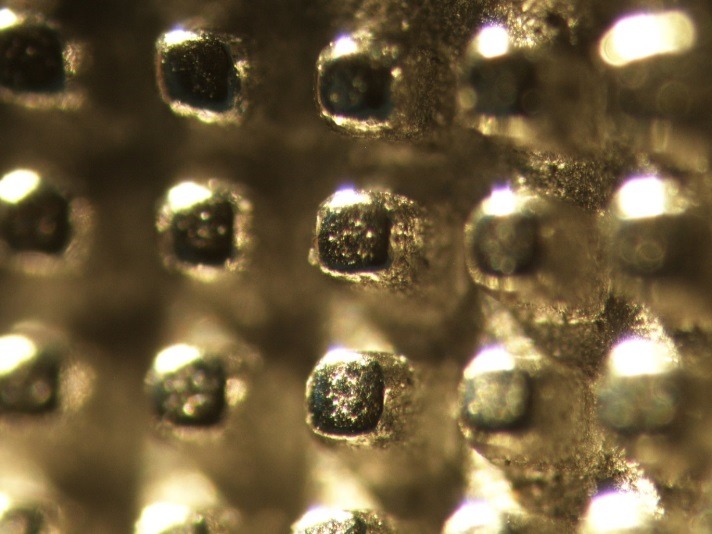
Anchor-pylon base design at ×40 magnification.

Two types of metal brackets were used in this study: Brackets with mesh base design (ODP metal brackets with Accu-Lock mesh, Franklin, IN, USA) illustrated in Figure 2, and brackets with anchor pylons illustrated in Figure 3 (ODP metal brackets with Anchor-Lock pad, Franklin, IN, USA). The only difference was the design of the bracket base.The teeth in one group were bonded using brackets with mesh base design, while in the other group they were bonded using brackets withanchor-pylon base design.

### Bonding

All the instruments used in this study were brand-new. Before bonding, the buccal surfaces of the teeth were cleansed with fluoride-free pumice and water andthen dried for 10s by air spray.^5^

The buccal surfaces of the samples were etched with 37% phosphoric acid gel (3M/Unitek, Monrovia, USA) for 15s,rinsed with water spray for 10s and dried with air spray until chalky appearance of enamel was observed. After application of a thin layer of Transbond XT Primer-Adhesive (3M/Unitek, Monrovia, USA) on the enamel,^5,13^ Transbond XT (3M/Unitek, Monrovia, USA) light-cured adhesive was applied on the bracket base and placed mesio-distally and occluso-gingivally at the center of the buccal surface on the long axis of the crown firmly by one orthodontist until a tight contact was achieved.^8,14^

The adhesive was light-cured with the use of a light-curing unit for 10s on the mesial aspect and for 10s on the distal aspect of the bracket.^13^

### Debonding

Formaximal bond strength, the samples were stored in distilled water and debonded 7 days after bonding.^17^ The brackets were debonded by one orthodontist using Weingart pliers, squeezing the mesial and distal wings.^8^

Then ARI scores were evaluated under a stereomicroscope at ×10. ARI for each sample was scored as follows:^18^

1: All the adhesive remaining on the tooth

2: More than 90% of the adhesive remaining on the tooth

3: 10‒90% of the adhesive remaining on the tooth

4: Less than 10% of the adhesive remaining on the tooth

5: No adhesive remaining on the tooth

**Figure 4:**
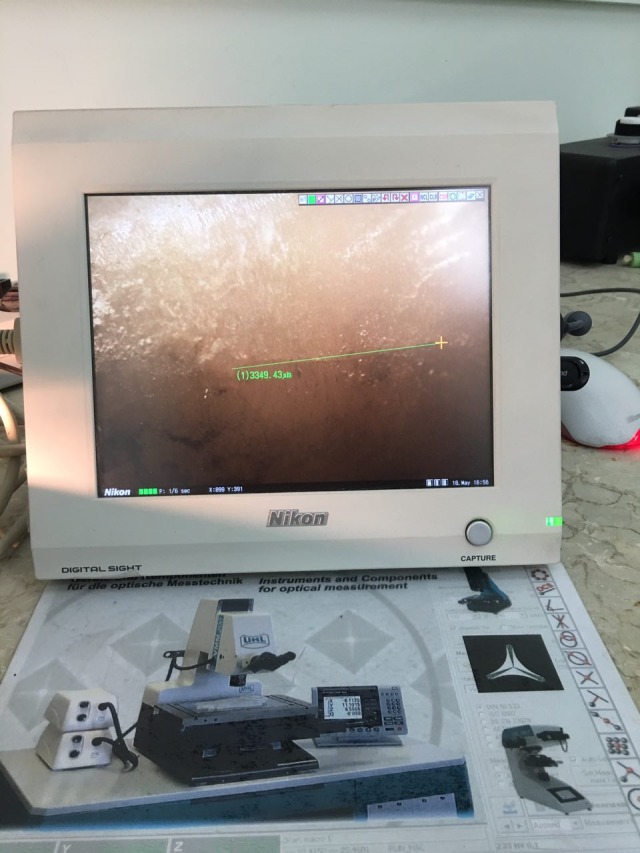
Crack length determination using optical stereomicroscope and digital camera.

In the next step, the remaining adhesive was removed with a 12-bladed carbide bur on a low-speed handpiece at 20000 rpm without water cooling. Then the samples were cleansed by rinsing in water.^14^ Two orthodontists observed and calculated the number and length of cracks under a stereomicroscope at ×40, which was connected to a digital camera capable of linear measurements. ICC was calculated at 90% between the observers, indicating good agreement (Figure 4).,^20^

**Figure 5:**
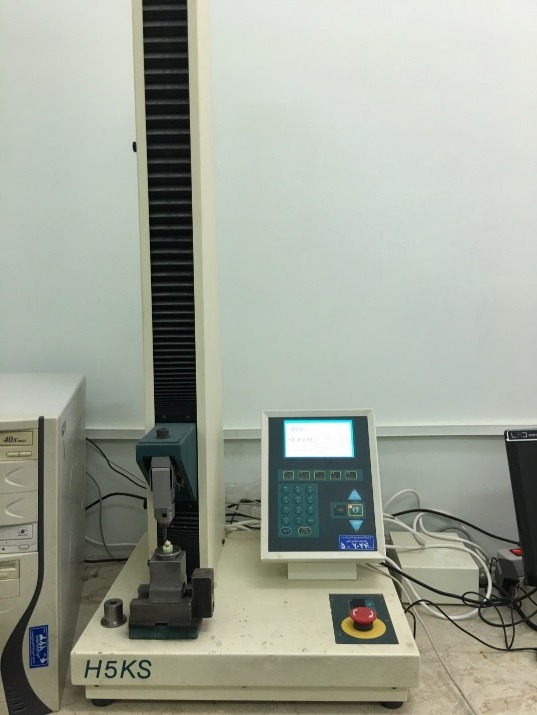
Universal testing machine.

The authors purposed evaluation of the probability that the selected brackets with different base design might have different shear bond strength. Forty teeth similar to original samples were collected and divided into two groups (N=20) randomly, and bonded by the same procedure. Teeth in one group were bonded using mesh base design brackets and in the other group were bonded using anchor-pylon base design brackets. Shear bond strength was calculated through a universal testing machine (Hunsfield Test Equipment, H5K-S model, England) at a crosshead speed of 1 mm/min for debonding the brackets (Figure 5).,^22^

### Statistical analysis

Kolmogorov-Smirnov test was applied to evaluate normal distribution of data. For evaluation of ARI score differences between the two groups, chi-squared test was used. The number of cracks in each group before bonding and after debonding was compared using Wilcoxon test. Mann-Whitney U test was used to compare the number of cracks between the two groups. ANCOVA was used for crack length comparison after and before debonding in each group and between the two groups. Crack length mean adjustment before bonding was performed with independent t-test. Independent t-test was used for shear bond strength comparison. Statistical significance was setat P<0.05.

## Results

Table 1 presents distribution of adhesive remnant index (ARI) scores for anchor-pylon base design group and meshgroup. Chi-squared test revealed significant differences in ARI scores between the two groups (P=0.014). The most prevalent ARI scores for anchor-pylon base andmesh base were 4 and 2, respectively.

**Table 1:** Distribution of ARI scores in two types of bracket

	**ARI Index**				
	**1**	**2**	**3**	**4**	**5**
**Brackets with mesh base**	1 (2.3%)	0	23 (52.3%)	11 (25%)	9 (20.5)
**Brackets with anchor pylons**	0	0	10 (22.7%)	14 (31.8%)	20 (45.5%)

Mean crack numbers, including mean±SD before bondingfor mesh base design was 2.59±1.51, with 3.68±1.47after debonding. For anchor-pylon base, mean crack numbers ±SD before bonding was 2.63±1.43, with 4.43±1.77 after debonding. According to Wilcoxon nonparametric test, the increase in the number of cracks in each group was significant after debonding (P<0.001). Mann-Whitney U nonparametric test revealed no significant differencesbetween the two groups before bonding (P=0.95) and after debonding (P=0.07) as presented in Table 2.

**Table 2:** Comparison of crack numbers between two types of brackets before bonding and after

		**Number**	**Mean**	**Standard deviation**	**P-value**
**Before**	**anchor-pylon**	44	2.63	1.43	0.95
	**Mesh**	44	2.59	1.51	
**After**	**anchor-pylon**	44	4.43	1.77	0.07
	**Mesh**	44	3.68	1.47	

Independent t-test showed that before bonding there was no significant difference in enamel crack lengths between the two groups (P=0.09). ANCOVA revealed that considering adjustment of enamel crack length before bonding in the two groups, crack length mean increased significantly in each group and was significantly greater in anchor-pylon base group compared to the mesh group after debonding as illustrated in Table 3 (P<0.001).

**Table 3:** Crack length mean difference after debonding in each group

	**Number**	**Mean**	**Standard deviation**	**P-value**
**Crack length mean after debonding in mesh base**	44	2544.53µm	865.09µm	<0.001
**Crack length mean after debonding in anchor-pylon base**	44	2693.63µm	694.68µm	

Mean shear bond strengths for anchor-pylon brackets and mesh brackets arepresented in Table 4. Independent t-test showed that shear bond strength was significantly higher in anchor-pylon base group (P<0.001).

**Table 4:** Comparison of shear bond strength between the two groups

	**Number**	**Mean**	**Standard deviation**	**T-value**	**Degree of freedom**	**Mean difference between groups**	**P-value**
**Anchor-pylon base**	20	13.4MPa	1.29	14.31	38	5.89MPa	<0.001
**Mesh base**	20	7.51MPa	1.31				

## Discussion

A new kind of bracket base design offered by ODP (USA) Company is called anchor-pylon base.This base has miniature pylons for adhesive retention, which act like strong anchors that are firmly embedded in the adhesive. The company claims that it provides equal or greater retention than mesh bonding pads. The anchor pylons are engineered at an acute angle relative to the torque and provide undercuts for adhesive retention, whereas mesh pads use the common technology of 80-guage foil mesh for adhesive retention.

Studies conducted by Gibas et al,^11^ Wang et al^3^ and Bishara et al^9^ concluded that base design affectsshear bond strength. To confirm the shear bond strength difference between the groups that might justify differences in stress exertion on enamel during debonding, the research group employed other samples similar to the original samples for shear bond strength assessment in a universal testing machine. The results indicated significantly higher shear bond strength for brackets with anchor pylons (P<0.001), which supports the results of a study by Gibas et al.^11^

According to the findings of this study, the two different base designs exhibitedsignificant differences in the distribution of ARI scores.ARI score distribution between the two groups showed that in theanchor-pylonbase design bond failure occurred at enamel‒adhesive interface in half of the samples whereas only 20% of mesh base design brackets showed this undesirable bond failure mode and overall, less adhesive remained on the tooth after debonding. Considering the same bonding material, bonding procedure, debonding procedure and randomization, it seems rational to assume that enamel bonding area to adhesive were similar in the two groups. More bond failure between the adhesive and enamel in the anchor-pylon base group is a manifestation of greater retention of anchor-pylon base design to adhesive, which leads to more stress transmission to enamel during debonding.

Bishara et al^9^ reported thatin mesh-base design brackets, including single-mesh base and double-mesh base design, most of the adhesive remained on the tooth surface, confirming the results of the present study. Gibas et al compared ARI scores between sandblasted anchor-pylon base brackets and sandblasted mesh-base brackets and concluded that brackets with anchor-pylon base design left less adhesive on the tooth surface compared to mesh-base design brackets and were more retentive.^11^ The results of the current study support the findings above.

The number and length of enamel cracks in each group increased significantly after debonding, consistent with other studies in this respect. This can be attributed to debonding stress on enamel or the need for the use of adhesive removal instruments.^16,23-25^

The reason why stereomicroscope was used to determine crack length and number and why methods like X-EDS (X-Ray Energy Dispersive Spectroscopy) were not employed was that most of the studies using X-EDS for surface damage and crack evaluation employed destructive methods and sectioning of teeth.^26,27^ Also, in this study the aim was comparison of enamel cracks before and after debonding, so we needed intact samples to bond and debond. Similar studies have employed the same method of optical microscopy for crack evaluation.^16,20^ We know the limitations of this method for evaluating the exact dimensions of enamel cracks, but due to comparative nature of this study, the error seems to be similar in each group and the comparison itself matters‏.

Due to higher shear bond strength in anchor-pylon base brackets and more destructive bond failure mode in this group, greater increase in enamel crack numbers after debonding was anticipated.^28,29^ Contrary to our prediction, the results demonstrated that enamel crack numbers increased to a greater degree in the anchor-pylon group but this increase was not statistically significant. This might be attributed to the existence of primary cracks before bonding that can serve as stress accumulation regions and may influence developing of new cracks as a confounding factor in both groups. Another reason for this finding might be the greater need for use of rotary appliances for removal of remnant adhesive in the mesh-base group. This in turn damages the enamel and compensates the lower stress during debonding and leads to an increase in the number of cracks as well.^30^ There were some differences between method used in the current study and the study conducted by Gibas et al.^11^ They compared shear bond strength in anchor-pylon base brackets and mesh-base bracketsand concluded that higher bond strength in anchor-pylon base brackets led to more enameldamage than mesh-base brackets.

We found significantly greaterincrease in enamel crack length in the anchor-pylon base group after debonding compared to the mesh group. This increase might be attributed to the bond failure mode as well as higher shear bond strength. Gibaset al also reported higher bond strength in anchor-pylon base brackets than mesh design brackets. These factors could influence enamel surface stress reception and damage, which partly confirms longer enamel cracks in the anchor-pylon base bracket group afterdebonding.^28,29^

Greater debonding stress in anchor-base brackets was manifested as an increase in crack lengththanin crack numbers. This might be attributed to the presence of prior enamel cracks before bonding that act as stress accumulation sites that might affect stress-induced damage.

Given the introduction of different bracket base designs to the orthodontic market, further studies are required to help orthodontists balance bond strength demands and iatrogenic side effects of debonding.

## Conclusion

1. Bracket base design can affect the bond failure mode.A greater amount of adhesive remained on enamel surface after debonding in mesh-base brackets compared to anchor-base brackets in which bond failure frequently occurredat enamel?adhesive interface, indicating a more retentive base design.
2. The number of new cracks formed after debonding was not significantly different between the two groups, yet base design affected enamel crack length increase after debonding, which was higher inbrackets with anchor-pylon base compared tobrackets with mesh-base.
3. Anchor-base design was more destructive than conventional meshbase design, resulting in more iatrogenic events during debonding.

## Acknowledgement

The authors would like to appreciate Dental and Periodontal Research Center, Tabriz University of Medical Sciences, for the financial and technical support of this study.

## Authors’ contributions

MHAA and PN designed the study; AHSH revised the manuscript. PN, MHAA, AHSH and SAA performed data acquisition and interpretation and manuscript drafting. All the authors have read and approved the final manuscript.

## Funding

This research was performed by financial support of the Dental and Periodontal Research Center, Tabriz University of Medical Sciences.

## Competing interests

The authors declare no competing interests with regards to the authorship and/or publication of this article.

## Ethics approval

This research was approved by Research Ethics Committee of Tabriz University of Medical Sciences in 2016.
